# Examining the Associations between Post-Stroke Cognitive Function and Common Comorbid Conditions among Stroke Survivors

**DOI:** 10.3390/ijerph192013445

**Published:** 2022-10-18

**Authors:** Helena W. Morrison, Melissa M. White, Janet L. Rothers, Ruth E. Taylor-Piliae

**Affiliations:** 1 College of Nursing, The University of Arizona, Tucson, AZ 85721, USA; 2 El Paso Veteran’s Administration Healthcare System, El Paso, TX 79930, USA; 3 BIO5 Institute Statistics Consulting Lab, The University of Arizona, Tucson, AZ 85721, USA

**Keywords:** community-dwelling, inflammation, mini-mental status exam, Montreal cognitive assessment

## Abstract

A considerable complication for stroke survivors is the subsequent development of cognitive decline or dementia. In this study, the relationship between the inflammation-centered comorbidity burden on post-stroke cognitive function among community-dwelling stroke survivors capable of independent living was examined. Data for this secondary analysis were collected from stroke survivors (n = 97) participating in a randomized clinical trial. Participants provided baseline responses, regarding cognitive function (mini-mental status exam, MMSE; Montreal cognitive assessment, MoCA), history of stroke comorbid conditions, and the Stroke Prognosis Instrument-II (SPI-II), an index of stroke comorbidity and recurrent stroke risk within the next two years. Relationships and differences between groups were tested for significance using Spearman’s correlation, Kruskal–Wallis, or Mann–Whitney U tests. Most stroke survivors (69%) had multiple comorbidities. Total SPI-II scores were negatively correlated to both MoCA and MMSE scores (r = −0.25, *p* = 0.01; r = −0.22, *p* = 0.03, respectively), and differences in MoCA scores among SPI-II risk groups (low, medium, high) were evident (*p* = 0.05). In contrast, there were no differences in MoCA or MMSE scores when comorbid conditions were examined individually. Lastly, no gender differences were evident in cognitive assessments. Our data support the premise that comorbidity’s burden impacts post-stroke cognitive decline, more than a single comorbid condition. Inflammation may be an important component of this comorbidity burden. Future studies that operationalize this concept will better illuminate the complex phenomenon of post-stroke cognitive decline for improved clinical rehabilitation modalities.

## 1. Introduction

The impact of stroke reverberates beyond that of the initial injury. Dementia is a post-stroke complication that is present among stroke survivors, but reports of its prevalence are variable in the literature. Roughly one to three in every ten stroke survivors develop dementia after their first stroke [1]. However, the dementia occurs slowly over time, with a time to diagnosis of about 4 years [2,3], a delay that reflects the insidious nature of this post-stroke complication. While post-stroke dementia associates strongly with infarct size and location; for a quarter of these patients, stroke-related dementia is also associated with vascular conditions, such as hypertension, dyslipidemia, and diabetes mellitus, that are comorbid with stroke [2,4]. Comorbid conditions are defined as secondary clinical conditions that are present in conjunction with a primary condition that influences the outcome of the primary condition [5,6].

Although vascular predictors of post-stroke dementia remain varied in population-based studies, evidence from pre-clinical settings suggests that brain healing is poor post-stroke, as a result of liquefaction, a leaky glial scar, and chronic inflammatory milieu [7,8], an aspect of post-stroke recovery that is underacknowledged. Compounding this process, ischemic stroke rarely occurs in an entirely healthy patient. Rather, stroke is accompanied by an exhaustive list of comorbidities (often thought of as risk factors), many of which share a common feature—systemic inflammation [9]. Inflammation and adaptive immune responses, both occurring in situ and presenting systemically, are a constant barrage to the post-stroke compromised brain [10,11,12,13,14], representing an additional factor contributing to the risk for dementia onset over time. Understanding the predictors of post-stroke dementia, whether it be a vascular risk profile that is triggered by stroke injury or the impact of chronic inflammation on an insufficiently healed brain, will inform us of prevention efforts and enable the targeted clinical management of these predictor(s).

**Figure 1:**
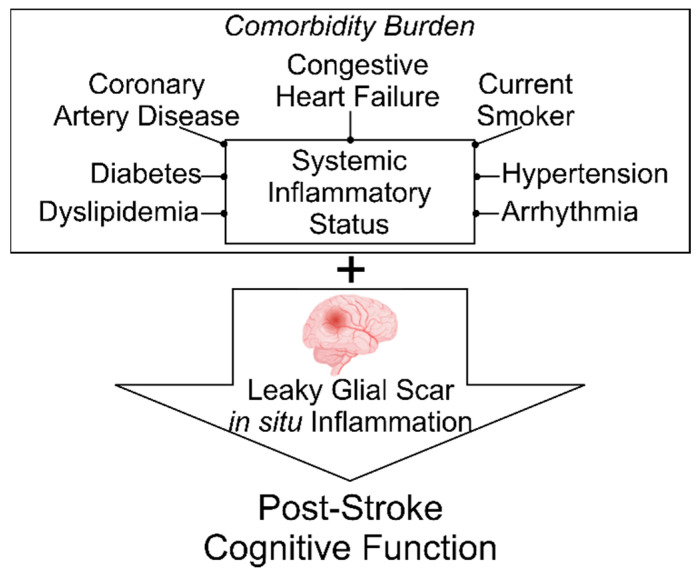
Framework guiding research inquiries examining relationships between stroke comorbid health conditions and post-stroke cognitive function. Ischemic stroke commonly occurs in the context of multiple co-occurring health conditions—comorbidity burden—and helps to define a person’s systemic inflammatory status. When stroke occurs, it occurs within this context. Compounding existing systemic inflammatory status, a leaky glial scar and in situ inflammation from poor brain wound healing occurs. Combined, these inflammatory states may contribute to or influence post-stroke cognitive function. (Figure developed, in part, with biorender.com, accessed on 30 September 2022).

The aim of this secondary data analysis was to examine the relationships between stroke comorbid health conditions and post-stroke cognitive function among community-dwelling stroke survivors capable of independent function. We hypothesized that the number of stroke comorbidities would be inversely associated with cognitive function, thereby suggesting that aggressive clinical management of the inflammation-centered comorbidity burden might delay or ameliorate the onset of post-stroke dementia. Figure 1 summarizes the framework guiding this investigation. 

## 2. Methods

### 2.1. Study Population

We analyzed interviewer-administered cognitive function assessment data from a randomized clinical trial (RCT) conducted by Taylor-Piliae and colleagues [15], carried out in a southwestern US city. In the original study, community-dwelling stroke survivors were recruited over a three-year period (January 2009 to January 2012) to participate in an intervention study to evaluate the effect of Tai Chi on physical function, fall rates, and quality of life [15]. Details about this study and recruitment strategies have been previously published [15]. Briefly, participants were recruited from Pima County, Arizona, through news media (e.g., newspaper, radio, and television), medical offices, outpatient rehabilitation centers, and community fitness centers. All study procedures in the original study were approved by the Institutional Review Board at the University of Arizona (approval #0800000257), and all participants provided written informed consent prior to data collection.

For this secondary data analysis, we utilized the de-identified baseline data from participants (n = 97) in the RCT, which included two cognitive function assessments. A portion of the original participants (n = 48) were excluded from the current analysis because cognitive assessments began after the RCT was initiated. This secondary data analysis study was approved by the Institutional Review Board at the University of Arizona (approval #1911170564).

### 2.2. Data Collection

The data analyzed in this study were collected in-person from consented participants in the original study, with the help of trained research assistants. These assistants administered the cognitive function assessments, queried participants about their medical history to ascertain stroke comorbidities, and were available to answer participant questions.

### 2.3. Cognitive Function Assessments

Cognitive function was assessed using the well-established and validated Montreal cognitive assessment (MoCA) test and mini-mental status exam (MMSE). The MoCA is a 30-point screening instrument categorized by eight cognitive domains, intended to be completed in 10 min. Each category is assigned higher points based on items that originally discriminated well with mild cognitive impairment [16]. The range for this instrument is 0 to 30. A score less than 26 indicates need for a referral for cognitive evaluation. For the MoCA instrument, in this population, the Cronbach α was 0.57, lower in this study, compared to the original validation study (0.83) [16] carried out in elderly (>70 years old) male and female Canadian participants with mild cognitive impairment, Alzheimer’s disease, and healthy controls.

The MMSE is an 11 question, 30-point screening instrument categorized by several cognitive domains. Each category is weighted based on domains responsible for cognitive aspects of mental functions [17]. The score range for this instrument is 0 to 30. Further evaluation is recommended for scores less than 25. Intended to differentiate psychiatric patients from those with organic dementia, the MMSE has been used extensively in Alzheimer’s disease, Parkinson’s disease, and age-related cognitive decline. Repeated administrations have been used to differentiate between normal age-related cognitive decline and neurodegenerative pathological processes. In this population, the Cronbach α for MMSE was 0.52. Lastly, the cognitive assessments MoCA and MMSE were significantly correlated (r = 0.62, *p* < 0.0001), indicating that, while both instruments are similar, they are not entirely duplicative.

### 2.4. Stroke Comorbidity Assessment

Data of participant conditions that are considered comorbid to their stroke recovery were collected through self-report methods. Participants were queried by a trained research assistant regarding their medical history, in addition to using the Stroke Prognosis Instrument-II (SPI-II). The SPI-II is an instrument used to assess the risk of stroke reoccurrence within two years of initial stroke, a practical self-report instrument used in outpatient study settings among study participants with non-disabling ischemic stroke (AUC 0.63) [18]. After tallying scores, participants were categorized, according to their SPI-II scores, as low, moderate, and high risk for recurrent stroke within two years, according to Kernan et al. (2000) cutoffs that were established by observing stroke outcome rates in the SPI-I study, using log-rank tests across risk groups [19]. Additionally, using regression analyses, the authors found that, by also including data on congestive heart failure and prior stroke history, the revised instrument further discriminated among the low, moderate, and high-risk groups [18].

### 2.5. Statistical Analysis

Data were analyzed using SPSS Version 27 (IBM SPSS Statistics for Windows, Armonk, NY, USA: IBM Corp.). Descriptive statistics for categorical variables were expressed as frequencies and relative frequencies. Because distributions of MoCA and MMSE scores were left skewed, descriptive statistics for these variables were expressed as medians with minimum and maximum scores, and relationships with these variables were assessed using non-parametric tests (Spearman’s correlation, Kruskal–Wallis test, Mann–Whitney U test). When appropriate, Bonferroni corrections were used for multiple comparisons.

## 3. Results

### 3.1. Self-Reported Health Conditions

In this secondary analysis, the mean age of these 97 participants was 68 ± 9.9 years (mean ± SD), 47% (n = 46) were female, and the predominant ethnicity was Caucasian (79.4%, n = 77). Individuals meeting recruitment criteria were functionally independent, with a mild to moderate functional disability score. This translated to a mean modified Rankin score of 2.1 ± 0.7, indicating that 73% (n = 71) of study participants were able to look after themselves without daily assistance. Additionally, in this data subset, the time passed since participants’ most recent stroke was 33 ± 50 months. Regarding cognition, 63% (n = 61) of participants had a MoCA score less than 26, and 5% (n = 4) had a MMSE score less than 25, values that necessitate a clinical referral for further cognitive evaluation. A majority of participants (69%, n = 67) had multiple comorbidities that accompanied their stroke survivor status; specifically, most participants had greater than 3 cardiovascular-related diseases. Participants’ self-reported health conditions that were comorbid with their stroke survivor status are reported in Table 1.

### 3.2. Correlations between Cognitive Function and Stroke Comorbidity

In this data set, significant relationships between the SPI-II total score and the cognitive function assessments were examined. We found that the SPI-II total scores were negatively correlated with both the MoCA and MMSE scores (r = −0.25, *p* = 0.01 and r = −0.22, *p* = 0.03, respectively). Since the SPI-II score is largely a function of the number and severity of comorbidities, this finding supports our hypothesis that higher comorbidity burden is associated with lower post-stroke cognitive function. Table 2 and Table 3 show the relationships between the SPI-II scores and cognitive function scores by cognitive domain. The SPI-II total score correlated significantly with one specific cognitive domain in the MoCA—recall words—and with very little association with other domains in either cognitive assessment tool. This negative correlation between SPI-II score and recall words domain score indicates that, as the risk of recurrent stroke increased, one’s ability to recall a list of five words without any cue tended to decline. In addition, in this population, the MoCA may have been more sensitive to determining these associations than the MMSE, as illustrated by the low percentage (5%), less than 25, triggering cognitive referral.

### 3.3. Cognitive Function According to Recurrent Stroke Risk Score

**Figure 2:**
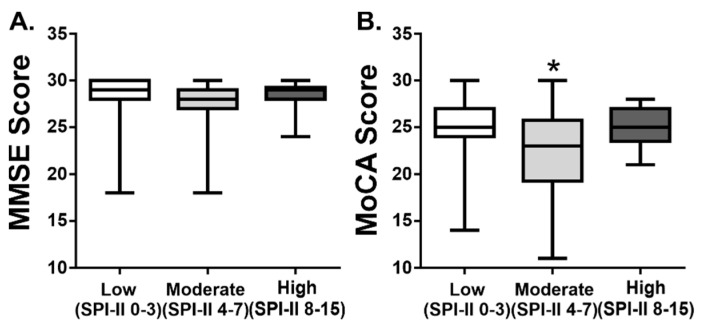
Cognitive function scores according to SPI-II category. Summary data of (**A**) MMSE and (**B**) MoCA scores, according to SPI-II rank: low (0–3), moderate (4–7), and high (8–15). Data are represented as boxplots with mean, min, and max values. Difference in MoCA scores were observed between SPI-II low and moderate ranks (Dunn’s post-hoc, * *p* < 0.05).

The differences between overall cognitive function scores and SPI-II groupings, low, moderate, or high, were examined. Data reported in Figure 2 summarize these differences. We found that there were no significant differences in MMSE scores, based on SPI-II groups (Kruskal–Wallis: MMSE: H = 4.07, *p* = 0.13). In contrast, there were significant differences in MoCA scores among SPI-II groups (Kruskal–Wallis: MoCA: H = 6.14, *p* = 0.05). In this case, those differences were noted between the moderate and low-risk groups (Dunn’s post-hoc test, *p* = 0.05, adjusted), such that the total MoCA scores were lowest (indicative of lower cognitive function) in the moderate-risk group, when compared to the low-risk or the high-risk groups.

### 3.4. Cognitive Function According to Stroke Comorbid Conditions

**Figure 3:**
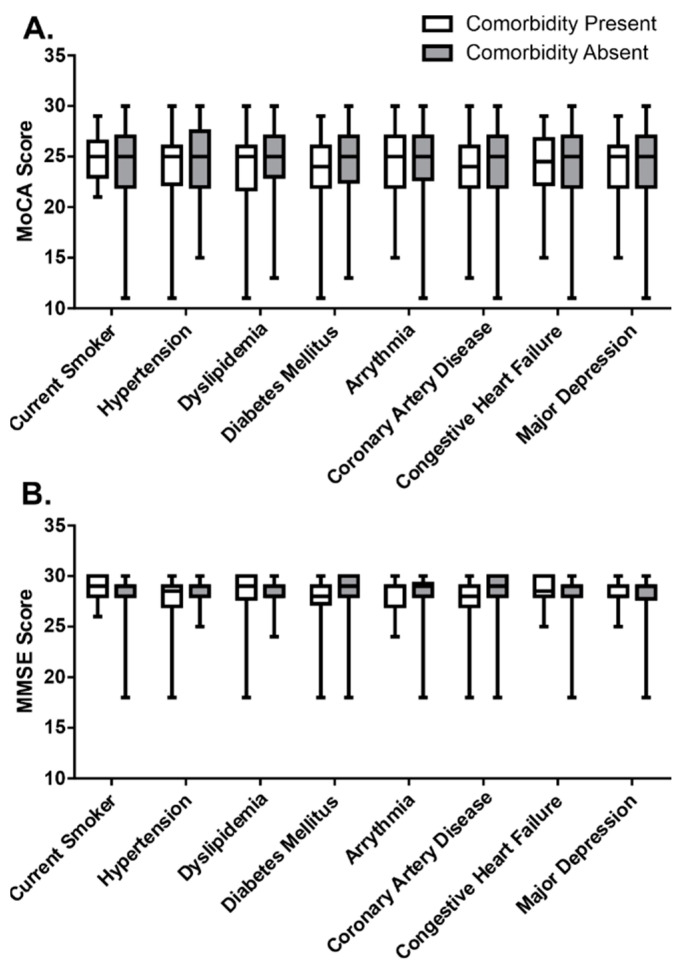
Differences in MoCA and MMSE score by stroke comorbid health conditions. Summary data of (**A**) MoCA and (**B**) MMSE scores in participants with and without comorbid condition. Data are represented as boxplots with mean, min, and max values; statistical testing via Mann–Whitney U test.

The relationship between an aggregate of prevalent cardiovascular self-reported comorbidities assessed in this study (e.g., diabetes mellitus, hypertension, dyslipidemia, and coronary artery disease) and the MoCA and MMSE scores were examined. This correlation demonstrated that, as the absolute number of comorbid conditions increased, there was a small decrease in cognitive function (Spearman’s, r = −0.128, *p* = 0.21 and r = −0.127, *p* = 0.22, respectively). There were no differences in MoCA or MMSE scores when the comorbid conditions were examined on an individual basis (Figure 3).

### 3.5. Cognitive Function and Gender

**Figure 4:**
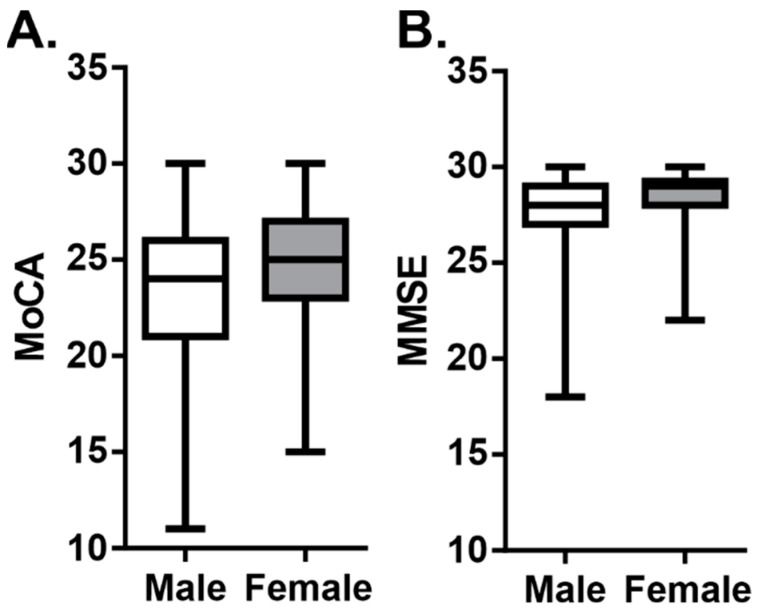
Cognitive function score, according to gender. Summary data of (**A**) MoCA and (**B**) MMSE scores in male and female participants. Data are represented as boxplots with mean, min, and max values; statistical testing via Mann–Whitney U test.

The effect of gender on post-stroke cognitive function was examined. Approximately half of the participants were male. Figure 4 summarizes the median scores for the total MoCA and MMSE scores, when stratified by gender. While females had slightly higher scores on both the MoCA and MMSE, compared to males, neither difference was statistically significant (Mann Whitney: Z = −1.37, *p* = 0.17; Z = −1.07, *p* = 0.28, respectively).

## 4. Discussion

The overall purpose of this secondary analysis was to evaluate the relationships between post-stroke cognitive decline and the presence of stroke comorbid conditions, as a group—comorbid burden—and on an individual basis. Secondarily, we evaluated the role of gender in post-stroke cognitive decline in this data set. Our primary finding was that, as the total SPI-II score increased (reflective of increasing number of comorbidities), the MoCA and MMSE scores decreased. These data support our hypothesis that, as the number of stroke comorbidities increased, this would be inversely associated with cognitive function. When the MoCA and MMSE scores were broken down by the contributing domain, the total SPI-II score was inversely, but not significantly, correlated with nearly all domains, however, with one exception—the MoCA recall domain. Additional data indicate that, when data were classified, according to SPI-II risk group, as low, medium, or high, there was no consistent change in either the MoCA or MMSE among these classifications. In addition, when participants were stratified by the presence or absence of singular comorbid conditions (e.g., those with and without HTN), the MoCA and MMSE scores were similar. Lastly, the MoCA and MMSE scores were not significantly influenced by gender.

Generally, the methods to assess comorbidity range from the simple task of summing the number of conditions present at the time of the primary event to including the weighted impact of each comorbid condition, creating an index. In the latter case, weight is often assigned according to a 1-year mortality value, as is found in the Charlson Comorbidity Index, Kaplan-Feinstein Index [20], or the Cumulative illness rating scale [21]. In other cases, comorbidity indices associate with, or are indexed to, function (e.g., functional comorbidity index) [22]. Therefore, while the burden of comorbidities, or comorbidity burden, is often associated with mortality, patient function, or the perceived impact of disease [5], few (with no investigations related to stroke) associate comorbidity burden with inflammation [23]. Yet two common denominators of comorbidities that is often present at the time of stroke are inflammation and immune responses. Moreover, the immune and inflammatory responses are mechanistically linked to post-stroke complications, such as dementia [10].

In this secondary data analysis, the SPI-II was used to assess study participants for stroke comorbidity. However, in this case, comorbid conditions were weighted according to stroke risk, rather than mortality, function, or inflammation. While we found that no singular comorbid condition was associated with post-stroke cognitive decline, as measured by MoCA or MMSE, the total scores were associated with cognitive decline and, therefore, may be indicative of a cumulative, rather than singular, burden. This is not an entirely novel finding in the literature [24,25]; however, the perspective in the literature, when it comes to correlations between disease states and cognitive function, is quite narrow. For example, studies often illustrate the differences in cognitive outcomes, depending on singular inflammatory conditions, such as diabetes [26], metabolic syndrome [27], obstructive sleep apnea [28], and hypertension [14,29], rather than a collective burden of multiple comorbid conditions. Therefore, this study moves beyond viewing stroke patients as having singular comorbidities, which is less common than those with multiple comorbidities. On the other hand, Walker et al. (2019) illustrated the association between systemic inflammation and cognitive decline [30]. A measure of cumulative comorbidity burden, if it could be re-oriented to reflect the contribution of inflammation, may be more relevant to the human condition, in the context of post-stroke cognitive function or decline, than the often-studied, narrow singular disease perspective.

Gender is an important biological factor in stroke science and, therefore, included in this secondary analysis. Although we did not observe gender differences in MoCA or MMSE scores among the community-dwelling stroke survivors in this study, a discussion is warranted. Gender-related data, regarding post-stroke cognitive decline, are contradictory and may be confounded by several factors, such as study population/location, recovery interval prior to data collection (which varied greatly in this study), and singleness. For example, prior research indicates that women were more likely to have cognitive decline than men using the MoCA instrument at 6-months post-stroke [31]. In contrast, the inverse was observed when measured four years post-stroke, with the male gender being associated with worsening cognitive decline [32]. In rural China, cardiovascular and social predictors are similar between genders; however, men become more susceptible to cognitive decline after the loss of a spouse [33]. Singleness, as an independent predictor for cognitive decline among men, is seen consistently in other research [32] and may be a confounder to gender differences. Unfortunately, singleness was not available in this data set.

A secondary data analysis design is an inherent study limitation. Primarily, the present study was not powered to address the current study questions; we show many interesting relationships that were not statistically significant. Additional limitations include the lack of consideration of stroke sub-types, according to TOAST criteria, due to these data not being collected in the original study, a non-exhaustive list of comorbid conditions, and the absence of data regarding level of education. However, others have published in this area [34,35]. The location of brain injury was not noted in this study, which may be important, in relation to the MoCA/MMSE cognitive assessment domains (recall, etc.). Such limitations may also account for increased systemic error and contribute to lacking power. Because this was not a prospective study, inflammatory markers (e.g., C-reactive protein, cytokines IL-6, TNFα, or neurofilament light) were not assessed. Rather, our data suggests the need to include such markers, to enhance our current tools for assessing comorbidity burden. Lastly, the internal consistency of the two cognitive measures were lower than 0.70 and may have impacted the results obtained. Because the original study was a convenience sample, we can only generalize these data to similar community-dwelling stroke survivors in the southwest in the United States. A strength of this study was the inclusion of two cognitive instruments. While the MMSE has been the gold standard in detecting cognitive decline, early cognitive changes may be less identifiable [36].

## 5. Conclusions

Comorbidity burden is associated with post-stroke cognitive decline. Our data support the premise that comorbidity burden impacts post-stroke cognitive decline more than a single comorbid condition. Inflammation may be an important component of this comorbidity burden. The link between comorbidity burden and inflammation may be key to understanding post-stroke cognitive decline. Future research that operationalizes the concept of inflammation-centered comorbidity burden will advance the management of post-stroke rehabilitation from a cognitive perspective.

## Figures and Tables

**Table 1:** Stroke survivor self-reported comorbid health conditions.

Comorbid Health Condition	n	%
Current Smoker	8	8.2
Hypertension	76	78.4
Dyslipidemia	66	68.0
Diabetes Mellitus	28	28.9
Arrhythmia	27	27.8
Coronary Artery Disease	23	23.7
Congestive Heart Failure	16	16.5
Major Depression	15	15.5

**Table 2:** Correlations between MoCA domains and SPI-II total.

MoCA Cognitive Function Domains	Correlation with SPI-II Total Score #	*p*-Value *
Executive Functioning	−0.08	0.42
Naming	−0.05	0.62
Digit List	−0.03	0.74
Repeat Sentence	−0.05	0.60
Similarity	−0.16	0.11
Recall Words	−0.33	0.001
Orientations	−0.03	0.76

# Spearman’s Rho, * Bonferroni-corrected critical value (alpha) is 0.007 to account for multiple comparisons (0.05/7 domain comparisons).

**Table 3:** Correlations between MMSE domains and SPI-II total.

MMSE Cognitive Function Domains	Correlation with SPI-II Total Score #	*p*-Value *
Orientation	0.01	0.89
Registration	−0.07	0.50
Attention-Math	0.07	0.52
Attention-Spell	−0.13	0.19
Attention Score	−0.17	0.10
Recall Words	−0.21	0.04
Language	-	-
Repetition	−0.05	0.61
3-Stage Command	−0.07	0.47
Reading	-	-
Writing	−0.10	0.33
Copying	−0.06	0.55

# Spearman’s Rho, Language and reading, are noted (-) as responses did not vary, * Bonferroni-corrected critical value (alpha) is 0.005 to account for multiple comparisons (0.05/10 domain comparisons).

## Data Availability

The data presented in this study are available on request from the corresponding author. The data are not publicly available for privacy reasons.
